# DNA entry, exit and second DNA capture by cohesin: insights from biochemical experiments

**DOI:** 10.1080/19491034.2018.1516486

**Published:** 2018-10-20

**Authors:** Yasuto Murayama

**Affiliations:** a Chromosome Biochemistry Laboratory, Center for Frontier Research, National Institute of Genetics, Mishima, Shizuoka, Japan; b Department of Genetics, SOKENDAI (The Graduate University for Advanced Studies), Mishima, Shizuoka, Japan

**Keywords:** Cohesin, SMC complexes, sister chromatid cohesion, DNA-DNA interactions, biochemical reconstitution

## Abstract

Cohesin is a ring-shaped, multi-subunit ATPase assembly that is fundamental to the spatiotemporal organization of chromosomes. The ring establishes a variety of chromosomal structures including sister chromatid cohesion and chromatin loops. At the core of the ring is a pair of highly conserved SMC (Structural Maintenance of Chromosomes) proteins, which are closed by the flexible kleisin subunit. In common with other essential SMC complexes including condensin and the SMC5-6 complex, cohesin encircles DNA inside its cavity, with the aid of HEAT (Huntingtin, elongation factor 3, protein phosphatase 2A and TOR) repeat auxiliary proteins. Through this topological embrace, cohesin is thought to establish a series of intra- and interchromosomal interactions by tethering more than one DNA molecule. Recent progress in biochemical reconstitution of cohesin provides molecular insights into how this ring complex topologically binds and mediates DNA-DNA interactions. Here, I review these studies and discuss how cohesin mediates such chromosome interactions.

## Chromosome organization by SMC complexes

Genomic DNA is at least one thousand-times longer than the cell and, in eukaryotes, is packed into a cellular nucleus in interphase. During cell proliferation, this long genetic material is duplicated and further compacted in order to equally distribute it to daughter cells. While this intricate process is taking place, genomic DNA must still be accessible for transcription factors to allow precise gene expression, and consequently, timely cell cycle progression and cellular differentiation. Numerous nuclear proteins contribute in a cooperative manner to convert genomic DNA into regulatable, dynamic supramolecular structures called chromosomes [1,2]. Among them, the ring-shaped Structural Maintenance of Chromosomes (SMC) complexes play central roles in chromosomal organization across all domains of life [3–7]. In eukaryotes, there are three groups of SMC complexes: cohesin establishes sister chromatid cohesion, which ensures proper chromosome distribution during cell division; condensin promotes mitotic chromosome condensation; and the SMC5-6 complex was identified as a DNA repair complex, although its role in chromosome organization is poorly understood. Bacterial SMC complexes also ensure proper chromosome distribution to daughter cells during rapid cell proliferation. In addition to its mitotic roles, cohesin contributes to interphase chromosome organization and is thought to modulate the interaction of promoters with cognate enhancer elements [8,9]. At the molecular level, cohesin is thought to establish DNA-DNA interactions between sister chromatids and/or different stretches of DNA segments on the same chromosome.

Although their established mitotic roles are different, notable similarities between cohesin and condensin have been reported. In an early genetic study, yeast cohesin was identified as a factor functioning in both sister chromatid cohesion and chromosomal condensation [10]. Cohesin clusters in axial-like structures on mammalian chromosomes when the activity of Wapl, which promotes cohesin release from chromatin, is compromised in interphase [11]. These axis-like structures, named *vermicelli*, are reminiscent of the localization of condensin to mitotic chromosomes. In Wapl-deficient cells, long-range chromosomal contacts were increased, with concomitant loss of compartments [12]; this resembles the characteristic landscape of mitotic chromosomal contacts, which is organized by condensin [13,14]. Thus, under certain conditions, cohesin has the potential to organize similar axial structures as condensin. These observations imply that cohesin organizes interphase chromosome structures through a mechanistic principle that is similar to the one employed by condensin for mitotic chromosomes.

Based on the ring structure, cohesin has been proposed to encircle DNA inside its cavity [15–17]. Indeed, purified fission yeast and human cohesin have been demonstrated to topologically entrap DNA in an ATP-dependent manner [18–20]. Topological DNA binding has been suggested to be the common characteristic of other essential SMC complexes [21–23]. At least for cohesin and condensin, integrity of the proteinaceous ring structure is critical for their essential chromosomal functions [16,21,24]. Thus, understanding how SMC complexes operate their ring opening for DNA entry and exit, and how the rings eventually achieve DNA-DNA interactions, are of great importance for chromosomal biology.

## Topological DNA binding by cohesin

**Figure 1.**
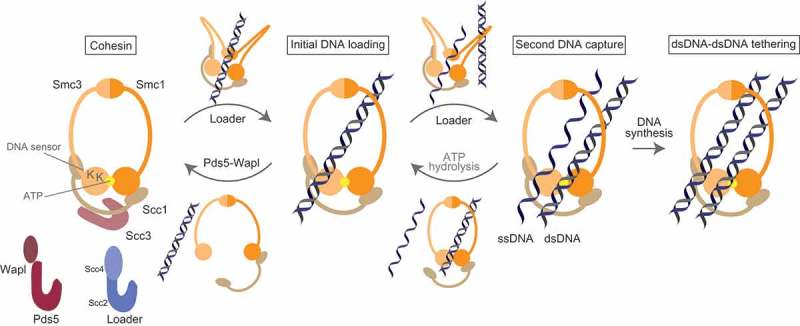
A model for DNA entry, exit and second DNA capture by cohesin. To highlight the actions of cohesin, only the trimeric ring (Smc1, Smc3 and Scc1) is shown throughout the figure. Topological DNA entrapment by cohesin might involve folding of the ring, which is facilitated by the loader via multiple cohesin contacts. Initially, DNA makes contact with the lysine DNA sensor of the Smc3 head. This contact triggers ATP hydrolysis, which enables DNA to pass through the SMC heads, leading to opening of the DNA transport gate. A similar route is used for DNA exit from cohesin. Upon ATP binding by cohesin, Pds5-Wapl facilitates abrogation of the Scc1-Smc3 interaction, resulting in DNA release. Second DNA capture is thought to be achieved by a repeat of the initial DNA loading. Cohesin initiates ring folding to make contact with the second DNA molecule, which must be single-stranded, and with the aid of the loader, cohesin is able to entrap this ssDNA while retaining the initial dsDNA. Second DNA capture is labile and apparently requires continuous ATP binding by cohesin. Once the weakly bound ssDNA is converted to dsDNA by DNA replication, cohesin establishes stable dsDNA-dsDNA interactions.

Cohesin is composed of four core subunits (Figure 1) [7]. The major part of the ring circumference is formed by a pair of SMC subunits, both of which are composed of a globular hinge domain connected to an ATPase head domain by 50 nm flexible antiparallel coiled-coils. Cohesin’s SMC subunits are Smc1 and Smc3, which form a stable heterodimer via hinge interaction. The ATPase heads also dimerize upon sandwiching two ATP molecules using the ATP Binding Cassette (ABC) signature motif [25]. The heads are further bridged by asymmetric interactions of the Scc1 kleisin subunit: Scc1’s N-terminus interacts with the coiled-coil neck close to Smc3’s head whereas its C-terminus associates with the bottom of Smc1’s head [26]. In addition, Scc1 interacts with the HEAT repeat Scc3 subunit which regulates chromatin association of cohesin. Using its flexible middle domain, Scc1 also serves as a docking platform for the HEAT repeat auxiliary proteins Pds5 and Scc2 [26–29]. Scc2, with its stable binding partner Scc4, promotes cohesin loading [30] whereas Pds5, with its substoichiometric binding partner Wapl, dissociates cohesin from chromatin [31–33].

If the molecular function of cohesin involves topological embrace, the ring must be opened for both DNA entrapment and release. In principle, this requires transient dissociation at one of three subunit interfaces: in between the Smc1-Smc3 hinges or at the interfaces between the SMC heads and the kleisin subunit (Smc3-Scc1 or Smc1-Scc1). As cohesin’s chromatin loading *in vivo* [34] and DNA entrapment in biochemical reconstitution systems [18] are ATP-dependent, opening of the DNA transport gate might be coupled with the actions of the SMC ATPase heads. In budding yeast, an Smc1 mutant protein that is defective in ATP binding (K39I, Walker A motif) failed to retain its stable interaction with Scc1 [35,36]. Under conditions where cohesin is able to bind ATP, the Smc3-Scc1 interaction can be disrupted by the concerted action of Wapl and Pds5 [37,38]. Similarly, ATP-dependent SMC-kleisin disengagement has been proposed for the bacterial Smc-ScpAB complex [39]. These findings suggest that protein interactions between the SMC heads and kleisin can be opened in the context of ATP usage.

Biochemical reconstitutions using fission yeast cohesin have found intrinsic similarities in both topological DNA loading and its release, proposing that cohesin uses similar DNA transport for both reactions (Figure 1) [37]. Both reactions require DNA sensing lysines of the Psm3^Smc3^ head ATPase, which would trigger head disengagement upon ATP hydrolysis and allow DNA transport at the head site into the small cavity formed by the SMC heads and Scc1. Upon ATP rebinding, SMC heads-kleisin interfaces would open to allow completion of DNA transport into the large cavity formed by the SMC subunits. In the loading reaction, the Mis4^Scc2^-Ssl3^Scc4^ cohesin loader complex folds back cohesin via multiple protein contacts along the ring’s circumference and exposes DNA sensing lysines to facilitate initial DNA contact [18]. In this context, the SMC heads function as an interlock gate. The notable point of this model is that the interlock gate could ensure a second round of DNA entry without loss of the initially entrapped DNA.

The same principle can also be applied to DNA release. DNA entrapped in the large cavity is transported to the small cavity upon ATP hydrolysis. Subsequent ATP rebinding, with the aid of Wapl and Pds5, triggers Psm3^Smc3^-Rad21^Scc1^ opening, resulting in DNA release. Ouyung et al. have shown that Pds5 interacts with Scc1’s N-terminal domain which includes the Smc3 binding interface; by occupying this interface, Pds5 physically prevents Scc1 binding to Smc3 [40]. These results suggest that Wapl initially destabilizes the Smc3-Scc1 interaction and Pds5 then keeps the DNA transport gate open. Crystallographic and bioinformatic studies have shown that Pds5 and Scc2, as well as Scc3, are paralogs and share similar U-shaped structures that are composed of stacked HEAT repeat helices [27–29,40]. Both Pds5 and Scc2 interact with the flexible middle domain of Scc1. Although their interaction motifs on Scc1 are different, these sites are located in close proximity to each other [27,41]. Interestingly, purified Pds5 competes with Mis4^Scc2^-Ssl3^Scc4^ for cohesin binding, suggesting that Scc2 and Pds5 bind to Scc1 in a mutually exclusive manner [37]. It is tempting to imagine that Scc2 opens the DNA transport gate through a mechanism that is similar to how Pds5 and Wapl operate.

Protein fusion and chemical crosslinking studies in budding yeast have proposed an alternative possibility for cohesin’s DNA entry [42]. Covalent fusions of Smc3-Scc1 or Smc1-Scc1 have only a minor impact on chromatin loading of cohesin or sister chromatid cohesion establishment. In contrast, hinge closing by insertion of rapamycin induced dimerization proteins, or replacing the hinge domains with unrelated dimerizing proteins, ablates the chromosomal functions of cohesin [42,43]. These findings suggest that the Smc1-Smc3 hinge interface operates as the DNA entry site. Studies from the *Bacillus subtilis* SMC protein argues for a different view of hinge contribution. Protein crosslinking at the ATPase heads completely prevented the *Bs*SMC dimer from binding to DNA, suggesting that head disengagement is part of the mechanism for efficient DNA binding of the *Bs*SMC dimer. Nonetheless, a positively charged cluster at the interface of the hinge domains, which faces the inside of the ring, also contributes to DNA binding [44]. Interestingly, a recent budding yeast study has demonstrated a critical role for positively charged patches at the hinge in initial DNA loading as well as topological DNA binding by cohesin [45]. This also leads to the proposal that the hinge domain functions as the initial DNA contact site for subsequent topological DNA entrapment. At the moment, *in vivo* protein engineering experiments and biochemical reconstitution studies have proposed different models for cohesin loading. It should be noted that both approaches have drawbacks: protein engineering might hinder cohesin’s action, including the initial DNA contacts in addition to closing subunit interfaces, whereas biochemical reconstitutions have not been able to test potential hinge opening directly. In conclusion, further functional investigations are needed to understand the precise DNA entry mechanism of the cohesin ring.

## DNA-DNA interactions: how to tether

Irrespective of the answer for DNA loading, cohesin is believed to mediate inter- and intramolecular DNA-DNA interactions. Early biochemical studies have already provided insightful observations for SMC’s DNA tethering ability. *Bs*SMC facilitates protein-DNA aggregation in an ATP-dependent manner. Curiously, this aggregation was only seen when single-stranded DNA (ssDNA) was used [46]. The *Escherichia coli* MukB SMC dimer mediates DNA-DNA tethering that is stimulated by ATP [47]. Similarly, the budding yeast Smc1-Smc3 dimer mediates DNA compaction, probably by forming DNA loops [48], whereas human cohesin has been shown to facilitate ligations between DNA strands by DNA ligase and DNA catenation by topoisomerase II [49].

If cohesin functions by topological embrace, how does the ring meditate DNA-DNA interactions? One scenario is that a single cohesin molecule embraces two DNA strands. This was originally proposed as the ring model to explain the mechanism by which cohesin establishes sister chromatid cohesion [3]. Protein crosslinking experiments at the interfaces of SMC and Scc1 subunits have also suggested that a single cohesin complex tethers replicated sister chromosomes in budding yeast cells [16,17]. In addition, single molecule observations have revealed that purified cohesin binds and diffuses along DNA as a single complex [19,20,50]. Another mechanistic explanation involves protein-protein interactions. DNA-DNA interactions can occur if two (or more) cohesin molecules, each of which holds a different DNA segment, physically interact with each other. Co-immunoprecipitation analysis has proposed direct cohesin-cohesin interactions in human cells [51]. In budding yeast, two different *scc1* alleles, each of which is nonfunctional, displayed interallelic complementation for cohesin loading on chromatin as well as establishment of sister chromatid cohesion [52]. These observations imply the existence of a protein interaction-based mechanism for DNA tethering. It is equally possible that potential cohesin-cohesin associations might occur during the process of topological DNA entrapment.

What mechanism enables a single cohesin complex to embrace two DNA strands? According to the interlock gate model, this would be possible if cohesin simply undergoes a second round of DNA capture while retaining the initial DNA inside the ring. We recently showed that purified fission yeast cohesin indeed can perform second DNA capture [53]. After achieving initial loading, DNA-bound cohesin catches the second DNA in a topological manner. Second DNA capture shares a series of biochemical similarities with the initial loading reaction, requiring ATP, the Mis4^Scc2^ cohesin loader and the DNA sensing lysines on the Psm3^Smc3^ head. This suggests that second DNA capture occurs through a repeat of the initial loading reaction (Figure 1). The critical difference is that second DNA capture was seen only when the second DNA was single-stranded (ssDNA). In contrast to stable entrapment of dsDNA, ssDNA capture is labile and apparently requires continuous engagement of SMC heads by ATP binding. However, once ssDNA is converted to dsDNA by DNA synthesis, cohesin can then retain stable DNA-DNA tethering. Cohesin also showed strict dsDNA to ssDNA order in the reaction; dsDNA-bound cohesin is capable of efficient second ssDNA capture but not vice versa.

The mechanism by which ssDNA facilitates second DNA capture is still unknown. Although such regions are limited in the genomic DNA, ssDNA regions are generated during the processes of DNA replication, repair and transcription. *In vivo*, ssDNA regions are readily coated by replication protein A, the trimeric ssDNA binding complex in eukaryotes [54]. Curiously, RPA inhibited second DNA capture by cohesin in biochemical reconstitution systems. This inhibitory effect was alleviated when an RPA mutant protein with reduced DNA binding ability was used, suggesting that RPA inhibits second DNA capture by sequestering ssDNA. We also found that RPA has a negative impact on the establishment of sister chromatid cohesion in budding yeast. The defects observed in cohesion establishment mutants (*ctf18* and *chl4*) were partially suppressed by introducing the aforementioned RPA mutation, whereas overexpression of RPA itself caused a substantial cohesion defect [53]. These findings are consistent with the idea that ssDNA is a potential target for DNA tethering by cohesin, at least for sister chromatid cohesion.

Other SMC complexes have also been reported to physically and functionally interact with ssDNA. Condensin is recruited to highly transcribed genes, probably by targeting unwound DNA segments [55]. The RPA mutation that suppresses the cohesion defect was originally identified as suppressor of a temperature-sensitive condensin mutant in fission yeast [56]. Similar to eukaryotic condensin, the *Bs*SMC complex is recruited to the rDNA region in a transcription-dependent manner [57]. *In vitro*, SMC5-6 complex preferentially binds to ssDNA [58]. *Bs*SMC also has higher affinity for ssDNA [46]. The MukB dimer is capable of topological DNA binding and also has a higher preference for ssDNA [59]. Taken together, these findings suggest that ssDNA has important biological functions in DNA-DNA tethering by cohesin and other SMC complexes.

## Implications for sister chromatid cohesion

Cohesin is recruited to chromatin in late G1 in budding yeast and in telophase in mammalian cells. This recruitment depends on cohesin’s ATPase and the Scc2-Scc4 cohesin loader [30]. At the same time, cohesin is susceptible to chromatin releasing activity mediated by Pds5-Wapl [31,32]. Thus, cohesin displays dynamic chromosomal association through the balancing acts of these auxiliary proteins. Soon after DNA is replicated, cohesin establishes physical connections between the newly formed sister chromatids [60]. Establishment of stable cohesion further requires acetyl modifications on the DNA sensing lysines of the Smc3 head by the acetyltransferase Eco1 [61–64]. This acetylation counteracts Wapl-driven cohesin release, probably by direct inhibition and/or recruitment of antagonizing factors such as sororin [37,65]. As a result, cohesion is maintained until the onset of anaphase. Finally, separase cleaves Scc1 at the middle of the linker region and cohesin is released from chromatin, which initiates equational chromosome segregation [24].

**Figure 2.**
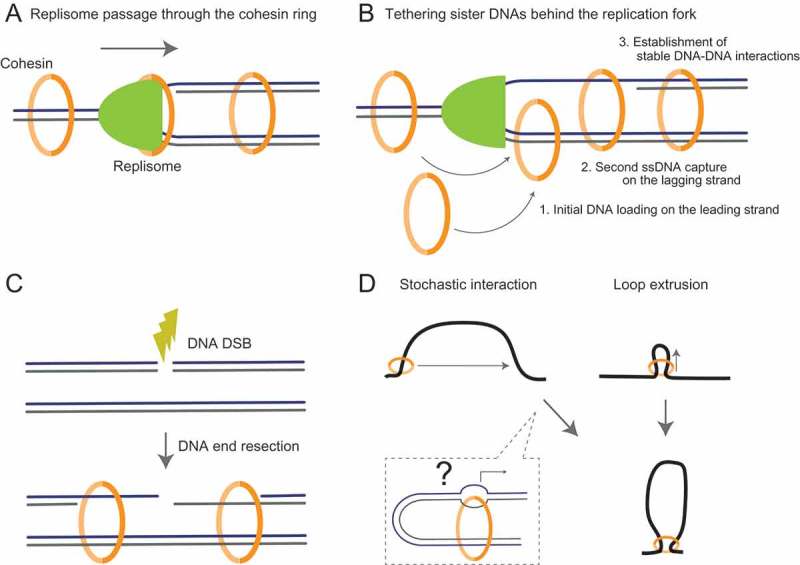
Possible models for establishment of chromosomal interactions by cohesin. Schematics represent establishment of sister chromatid cohesion in different contexts (a ~ c) and intrachromosomal loop formation (d). (a) In the replisome passage model, cohesin allows the replication machinery to pass through the inside of the ring, resulting in the embrace of two sister DNAs. (b) If cohesin cannot accommodate the replisome, it tethers two sister DNAs in the wake of the fork. In this context, the second DNA capture mechanism might be involved. Cohesin is initially loaded onto the leading dsDNA strand, followed by second capture with ssDNA on the lagging strand. Finally, lagging DNA synthesis converts the fragile dsDNA-ssDNA tethering into stable dsDNA-dsDNA cohesion. (c) Sister chromatid cohesion is also established upon DNA DSB formation. The break sites are processed by the concerted actions of nucleases including Mre11, generating ssDNA overhangs. Cohesin on the intact sister catches this ssDNA, leading to tethering of two sister DNAs in the vicinity of the DSB. (d) DNA loops can be formed by stochastic interactions between two distal DNA segments. Cohesin might target unwound ssDNA formed during transcription. Alternatively, cohesin forms the loop by extruding DNA through active and/or passive mechanisms.

How does cohesin connect newly replicated sister DNAs at the molecular level? As cohesin is already recruited to chromatin before S phase, the ring inevitably encounters the replication machinery as it progresses along DNA. One model predicts that cohesin permits replication fork passage through the inside of its ring. This passive mechanism finally allows a single cohesin to accommodate two replicated DNAs (Figure 2(a)) [66]. In budding yeast, the loader becomes dispensable for cell viability when inactivated just after, but not before, G1 phase, suggesting that the cohesin loaded onto G1 chromatin is capable of cohesion establishment. Interestingly, recent single molecule observations have suggested that the replication fork can proceed over chromatin-bound cohesin in *Xenopus* egg extracts [19].

An alternative possibility, but one that is not mutually exclusive with the fork passage model, is that cohesin actively tethers two sister DNAs in the wake of a replication fork. In this case, cohesin needs to distinguish the replicated sister DNA to avoid inaccurate DNA tethering (e.g., of a non-sister chromatid). The second DNA capture mechanism fits well with this scenario (Figure 2(b)) [53]. On the leading strand, DNA unwound by the replicative helicase is continuously converted to dsDNA whereas lagging strand synthesis is discontinuous, leaving ssDNA gap behind the fork prior to Okazaki fragment synthesis [67]. Cohesin, with the aid of the cohesin loader, might utilize the proximity of sister DNAs behind the fork: it is recruited to dsDNA on the leading strand and catches onto ssDNA on the lagging strand. Okazaki fragment synthesis converts this labile ssDNA capture and finally establishes stable DNA-DNA cohesion. The labile nature of ssDNA binding might allow correction of inaccurate second strand capture. In contrast, initial dsDNA loading ensures stable cohesin behind the fork and provides a period of opportunity for second DNA capture. This model postulates that cohesin is recruited to the replication fork. Indeed, it has been demonstrated that cohesin colocalizes with replication forks in budding yeast [68] and is recruited to replication forks in *Xenopus* egg extracts and human cells [69,70]. In addition, cohesin also interacts with the fork-associated Chl1 helicase [71]. Surprisingly, and in contrast to *ctf18* and *chl4*, the RPA mutation did not suppress the cohesion defect of the *chl1* mutant. This suggests that Chl1 might have a role in second DNA capture in the context of replication forks.

In addition to the replication fork, sister chromatid cohesion is also established at DNA double-strand breaks (DSBs) [72,73]. DSBs are the most deleterious form of DNA damage and are repaired by homologous recombination using, in most cases, a stretch of intact homologous DNA on the sister chromatid as a template [74]. The Mre11 nuclease, as part of the MRN complex, is the first detector of DSBs and initiates DNA resection in coordination with the actions of other nucleases and helicases, creating ssDNA overhangs at DSB sites [74]. This ssDNA region serves as a loading platform for the Rad51 recombinase to initiate homologous paring of the ssDNA and intact dsDNA template, which is followed by DNA synthesis [75]. Interestingly, cohesion establishment at DSBs depends not only on the Scc2-Scc4 loader, but also on Mre11, implying that ssDNA regions can be a target for second DNA capture by cohesin from the intact sister chromatid (Figure 2(c)) [72,73]. Thus, the second DNA capture mechanism provides a plausible explanation for establishment of DNA-DNA interactions between sister chromatids.

## Intrachromosomal interactions: another open question

Accumulating evidence from recent genome-wide chromosomal contact analyses have suggested that cohesin organizes intrachromosomal interactions. Two, non-mutually exclusive models have been proposed: cohesin stochastically tethers different DNA segments on the same chromosome (stochastic pairwise interactions) or the ring generates a chromatin loop by extruding a chromatin fiber via a passive or active mechanism (loop extrusions) [76,77]. Cohesin often establishes chromatin loops between enhancers and promoters [8]. Recent high resolution Hi-C studies have also found that cohesin makes contributions to interactions between enhancers and along gene bodies [78,79]. Substantial DNA helices can be opened during gene transcription, which would be potential targets for second DNA capture by cohesin (Figure 2(d)) [80]. Whether cohesin can establish DNA tethering in such a configuration is currently unknown. Recent *in vitro* single molecule observations have also opened the possibility that SMC complexes function as DNA loop extrusion machines. Purified budding yeast condensin promotes the formation and enlargement of DNA loops in an ATP-dependent manner [81]. In a different experimental setting, condensin has also been shown to translocate along DNA unidirectionally, which requires its intact ATPase [82]. These studies have suggested that condensin functions as an active machine to mediate DNA loop extrusion. Interestingly, although the cohesin loader stimulates cohesin’s DNA-dependent ATPase, cohesin itself is capable of topological DNA binding without efficient ATP hydrolysis [18,37]. This suggests that cohesin’s ATPase has other roles in addition to facilitating DNA loading [83]. However, in contrast to budding yeast condensin, ATP-dependent, unidirectional translocations have not been seen for both the fission yeast and human cohesins [19,20,50]. It should also be noted that the purified budding yeast condensin can tether two intermolecular DNA strands [82]. Thus, cohesin and condensin might be equipped with activities for both stochastic interactions and loop extrusions.

Taken together, the recent studies employing biochemical reconstitution have started to unveil the molecular characters of SMC complexes. The application of such approaches to SMC proteins now provide unprecedented opportunities towards achieving a molecular understanding of these mysterious chromosomal rings.
